# Myoferlin silencing inhibits VEGFR2-mediated proliferation of metastatic clear cell renal cell carcinoma

**DOI:** 10.1038/s41598-019-48968-7

**Published:** 2019-09-02

**Authors:** Hyo Jung An, Dae Hyun Song, Hyun Min Koh, Yu-Min Kim, Gyung Hyuck Ko, Jeong-Hee Lee, Jong Sil Lee, Jung Wook Yang, Min Hye Kim, Deok Ha Seo, Se Min Jang, Dong Chul Kim

**Affiliations:** 1 Department of pathology, Gyeongsang National University Changwon Hospital, Changwon, South Korea; 2 Gyeongsang National University School of Medicine, Jinju, South Korea; 3 Gyeongsang Institute of Health Science, Gyeongsang National University Hospital, Jinju, South Korea; 4 Department of pathology, Gyeongsang National University Hospital, Jinju, South Korea; 5 Department of urology, Gyeongsang National University Changwon Hospital, Changwon, South Korea; 6 Department of pathology, Konyang University Hospital, Daejeon, South Korea

**Keywords:** Biomarkers, Oncology, Urology

## Abstract

Recently, ramucirumab, a drug that targets vascular endothelial growth factor receptor (VEGFR), was clinically approved; therefore, we evaluated VEGFR2 expression and its predictive roles in tumor progression in clear cell renal cell carcinoma (CCRCC). Since we do not have many options for treating aggressive renal cell carcinoma patients, the application of anti-VEGFR2 therapy might be useful. Myoferlin (MYOF) is a 230 kDa transmembrane multi-C2-domain protein that contributes to plasma membrane repair, fusion, and endocytosis and is overexpressed in several invasive cancer cell lines, including breast, pancreas, and malignant melanoma. It forms a complex with VEGFR2 to inhibit VEGFR2 degradation. In this study, a total of 152 patients who had undergone nephrectomy for CCRCC were enrolled. Based on tissue microarray (TMA) blocks, the positive intensity and high proportion of MYOF showed a statistically significant correlation with the negative intensity (p < 0.001) and low proportion (p < 0.001) of VEGFR2, respectively. In addition, Fuhrman’s nuclear grade ≥3 showed a significant correlation with VEGFR2 expression. In multivariate analysis, CCRCC patients with positive MYOF and negative VEGFR2 expression demonstrated poor clinical outcomes. We confirmed that positive MYOF expression and negative VEGFR2 expression were positively correlated in this CCRCC population. Knocking down MYOF in Caki-1 cells resulted in the downregulation of VEGFR2 at both mRNA and protein levels. Wound healing assays revealed that the loss of MYOF in Caki-1 cells decreased cell confluence compared to that in control cells. We demonstrated that MYOF influences cellular proliferation of the metastatic CCRCC cell line by regulating VEGFR2 degradation. Combined therapies targeting the MYOF and VEGFR2 pathways might be effective against metastatic CCRCC to increase patient survival.

## Introduction

Vascular endothelial growth factors (VEGFs) are important factors that control angiogenesis, and currently, five types, namely, VEGF-A, VEGF-B, VEGF-C, VEGF-D, and PLGF (placental growth factor), have been identified. VEGFs bind to three VEGF receptors (VEGFR1, -2, and -3), which are receptor tyrosine kinases (RTKs), and redundantly to secondary receptors, such as heparin sulfate proteoglycans and neutropilins. The VEGF signaling pathway is associated with cellular proliferation and migration and endothelial cell permeability. Recently, ramucirumab, a receptor antagonist that targets VEGFR2, has come to light. By disrupting VEGF signaling, ramucirumab is known to inhibit the majority of the downstream effects of VEGF in angiogenesis. Ramucirumab is clinically approved and used in the treatment of advanced gastric cancer, gastroesophageal junction adenocarcinoma, non-small cell lung cancer, and metastatic colorectal cancer. Since we do not have many options for treating aggressive renal cell carcinoma patients, the application of anti-VEGFR2 therapy might be useful. Myoferlin (MYOF) is a 230 kDa protein containing a transmembrane domain and six C2 domains that contribute to plasma membrane repair, fusion, and endocytosis. MYOF has been found to regulate the cellular phenotype and the malignant capacity in various types of cancers. In melanoma cell line (A374), knockdown of MYOF suppressed vasculogenic mimicry via reducing matrix metalloproteinase -2 (MMP-2) and increasing mesenchymal-epithelial transition^1^. MYOF reduced cancer cell migration and invasion through regulating autocrine TGF-ß1 signaling in breast cancer cell line (MDA-MB-231)^2^. In pancreatic ductal carcinoma cell (Panc-1), MYOF silencing decreased cancer cell migration by reducing mitochondrial respiration^3^. Bernatchez *et al*. suggested that MYOF forms a complex with VEGFR2 in the cell membrane, and when MYOF expression is reduced, VEGFR2 is downregulated due to the disruption of membrane integrity^4,5^. Previously, we reported that the overexpression of MYOF was associated with poor clinical outcomes in clear cell renal cell carcinoma (CCRCC)^6^. However, there were limited numbers of patients with metastatic CCRCC represented in tissue microarray (TMA) blocks. Herein, we studied the influence of the VEGFR2 signaling pathway controlled by MYOF in Caki-1, a metastatic CCRCC cell line, and discussed the prospective efficacy of ramucirumab in patients with CCRCC.

## Results

### Patient characteristics

A total of 152 patients with CCRCC were enrolled in this study. The clinicopathological data for the CCRCC patients are summarized in Table 1. Among these patients, 109 were male. The mean age of the patients was 59.9 years (range: 32–83 years). The mean follow-up period was 4.33 years. Among the 152 patients with CCRCC, 25 suffered from a more advanced stage of disease, including lung metastasis, multiple organ metastases, bone metastasis, brain metastasis, liver metastasis, and local recurrence. The distribution of T stages was as follows: 1a: 91 (59.9%), 1b: 24 (15.8%), 2a: 9 (5.9%), 2b: 3 (2.0%), 3a: 2 (1.3%), and 4: 2 (1.3%). For Fuhrman’s nuclear grade, 26 (17.1%) cases were of grade 1, 102 (6.1%) were of grade 2, 19 (12.5%) were of grade 3, and 5 (3.3%) were of grade 4.

**Table 1:** Clinicopathological information of clear cell renal cell carcinoma patients.

Variable		Value (%) (n = 152)
Age, mean (range)		59.9 (32–83)
Sex (Male/Female)		109/43
Advanced RCC	Lung metastasis	9
	Multiple metastasis	6
	Bone metastasis	4
	Brain metastasis	2
	Liver metastasis	1
	Local recurrence	3
Follow up period, mean (years)		4.33
T stage	1a	91 (59.9%)
	1b	24 (15.8%)
	2a	9 (5.9%)
	2b	3 (2.0%)
	3a	21 (13.8%)
	3b	2 (1.3%)
	4	2 (1.3%)
Fuhrman’s nuclear grade	1	26 (17.1%)
	2	102(67.1%)
	3	19 (12.5%)
	4	5(3.3%)
VEGFR2 Intensity	Negative (0, 1+)	99 (33.0%)
	Positive (2+, 3+)	201 (67.0%)
VEGFR2 Proportion	low (<30%)	34 (11.3%)
	High (30%≤)	266 (88.7%)

Values are presented as numbers (%). Four cores for VEGFR2 of tissue microarray were not informative due to loss of the tissue specimen.

### MYOF, VEGFR2, and CD31 expression in CCRCC

**Figure 1:**
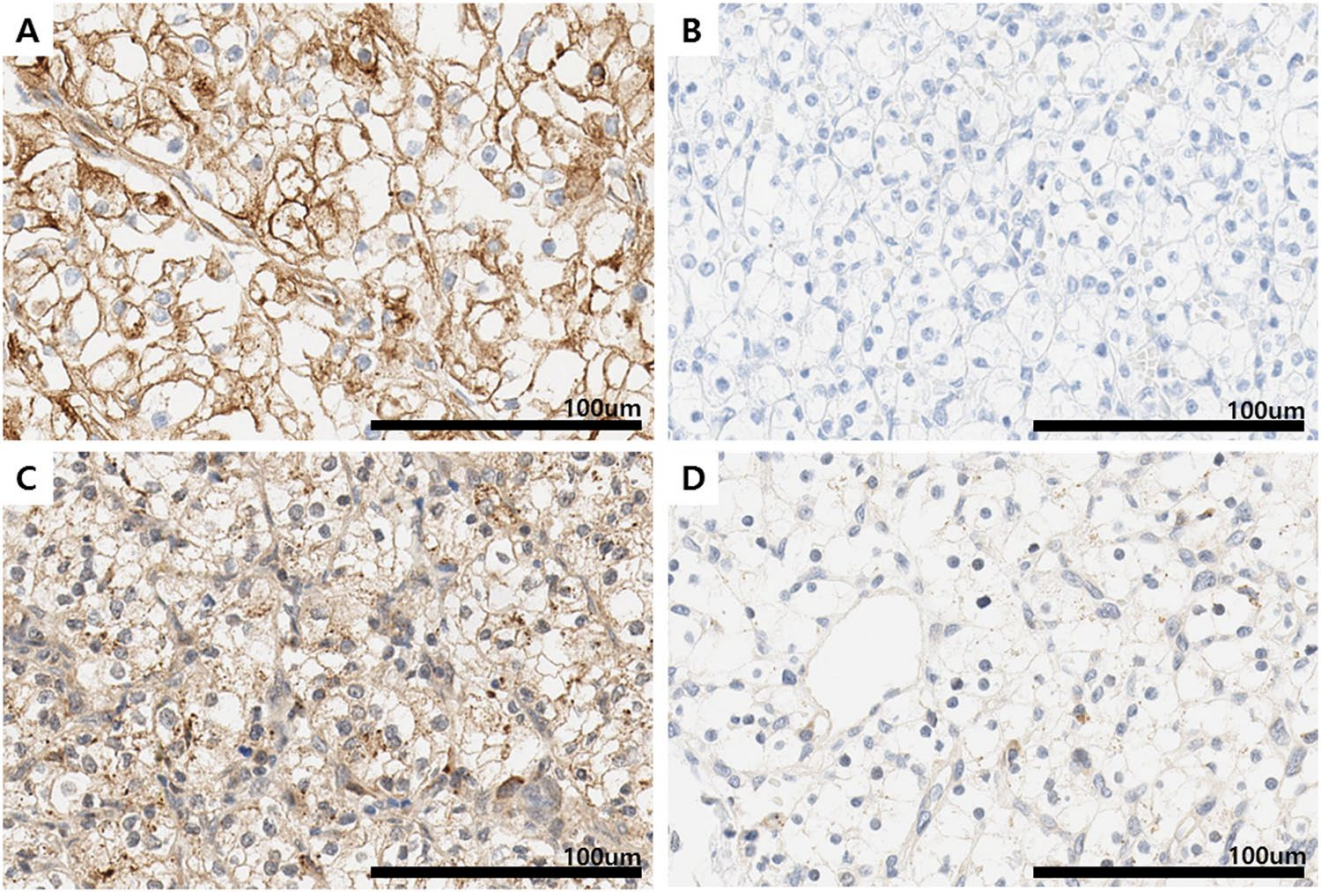
Representative images of Myoferlin and VEGFR2 staining patterns. **(A)** Strong positive and distinct membranous staining of MYOF in CCRCC (x200). **(B)** Negative expression of myoferlin (x200). **(C)** Strong positive and distinct membranous and cytoplasmic staining of VEGFR2 in CCRCC (x200). **(D)** Negative expression of VEGFR2 (x200).

With respect to the distribution of MYOF and VEGFR2 expression, staining intensities of only 2+ or 3+ were scored as positive. Among 304 cores, 214 (70.4%) were positive for MYOF, while 211 (66.1%) were positive for VEGFR2. With respect to the proportion of tumor cells expressing these markers, 40 (13.2%) samples were scored as having low and 264 (86.9%) were scored as having high MYOF expression. On the other hand, 34 (11.2%) samples were scored as having low and 266 (87.5%) as having high VEGFR2 expression. Images of immunohistochemical staining for MYOF and VEGFR2 are shown in Fig. 1. With respect to CD31 expression, staining intensities of 1+, 2+ and 3+ were scored as positive. Among 297 cores (except for 7 cores with tissue loss), 297 (100%) were positive for CD31.

### The relationship between MYOF and VEGFR2 expression and clinicopathological data for CCRCC

The positive intensity and high proportion of MYOF showed a statistically significant correlation with the negative intensity (p < 0.001) and negative proportion (p < 0.001) of VEGFR2, respectively. Among the clinical and pathological factors (age, sex, T stage, and Fuhrman’s nuclear grade), Fuhrman’s nuclear grade ≥3 showed a significant correlation with a high proportion of VEGFR2 (p = 0.025).

### The relationship between MYOF expression and the number of CD31-positive endothelial cells

The absolute number of CD31-positive endothelial cells per TMA core was automatically counted using the Genie analysis tool (Leica Biosystems, Wetzlar, Germany). The mean value of CD31-positive cells was 6691.02, and the median value was 5159.000 (range: 85.00–121998.00). The cut-off value for the number of endothelial cells was 5000 cells, and the absolute number was determined to be increased when the number of CD31-positive endothelial cells was over 5000 per TMA core. The positive intensity of MYOF and the number of CD31-positive endothelial cells were significantly correlated (p = 0.002) (Table 2). Images of the CD31-positive endothelial cells are shown in Supplementary Fig. 2.

**Table 2:** The relationship between MYOF expression and the number of CD31-positive endothelial cells. (N = 297).

		CD31-positive endothelial cells		Total
		Less than 5000 cells	More than 5000 cells	
MYOF intensity	Negative	30	59	89
	Positive	110	98	208
Total		140	157	p-value = 0.002

### MYOF and VEGFR2 expression and survival analysis

To identify an independent prognostic factor, we performed a multivariate Cox proportional hazards regression analysis. CCRCC patients with positive MYOF expression exhibited poor disease-free survival (hazard ratio: 3.556, 95% confidence interval: 1.184–10.684, *p*-value = 0.024) and poor disease-specific survival (hazard ratio: 2.777, 95% confidence interval: 1.031–7.482, *p*-value = 0.043). In addition, CCRCC patients with negative VEGFR2 expression demonstrated poor disease-specific survival (hazard ratio: 3.602, 95% confidence interval: 0.117–0.658, *p*-value = 0.004) (Table 3). We confirmed that positive MYOF expression and negative VEGFR2 expression were positively correlated in this CCRCC population.

**Table 3:** Multivariate Cox proportional hazards regression model of disease-free and disease-specific survival for CCRCC patients.

Variables	Disease-free survival			Disease-specific survival		
	HR	95% CI	p-value	HR	95% CI	p-value
Age (<59 vs ≥59	2.383	0.943–6.022	0.066	1.236	0.600–2.547	0.565
Sex (male vs female)	1.166	0.354–3.841	0.800	4.007	1.386–11.584	**0.010**
T stage (1–2 vs ≥3)	11.138	4.580–27.090	<**0.001**	10.857	4.418–26.684	<**0.001**
Grade (1–2 vs ≥3)	3.577	1.543–8.291	**0.003**	5.561	2.504–12.349	<**0.001**
MYOF intensity (+ vs −)	3.556	1.184–10.684	**0.024**	2.777	1.031–7.482	**0.043**
VEGFR2 intensity (− vs +)	2.069	0.933–4.585	0.073	3.602	0.117–0.658	**0.004**

### MYOF and VEGFR2 were identified in metastatic clear cell renal cell carcinoma

**Figure 2:**
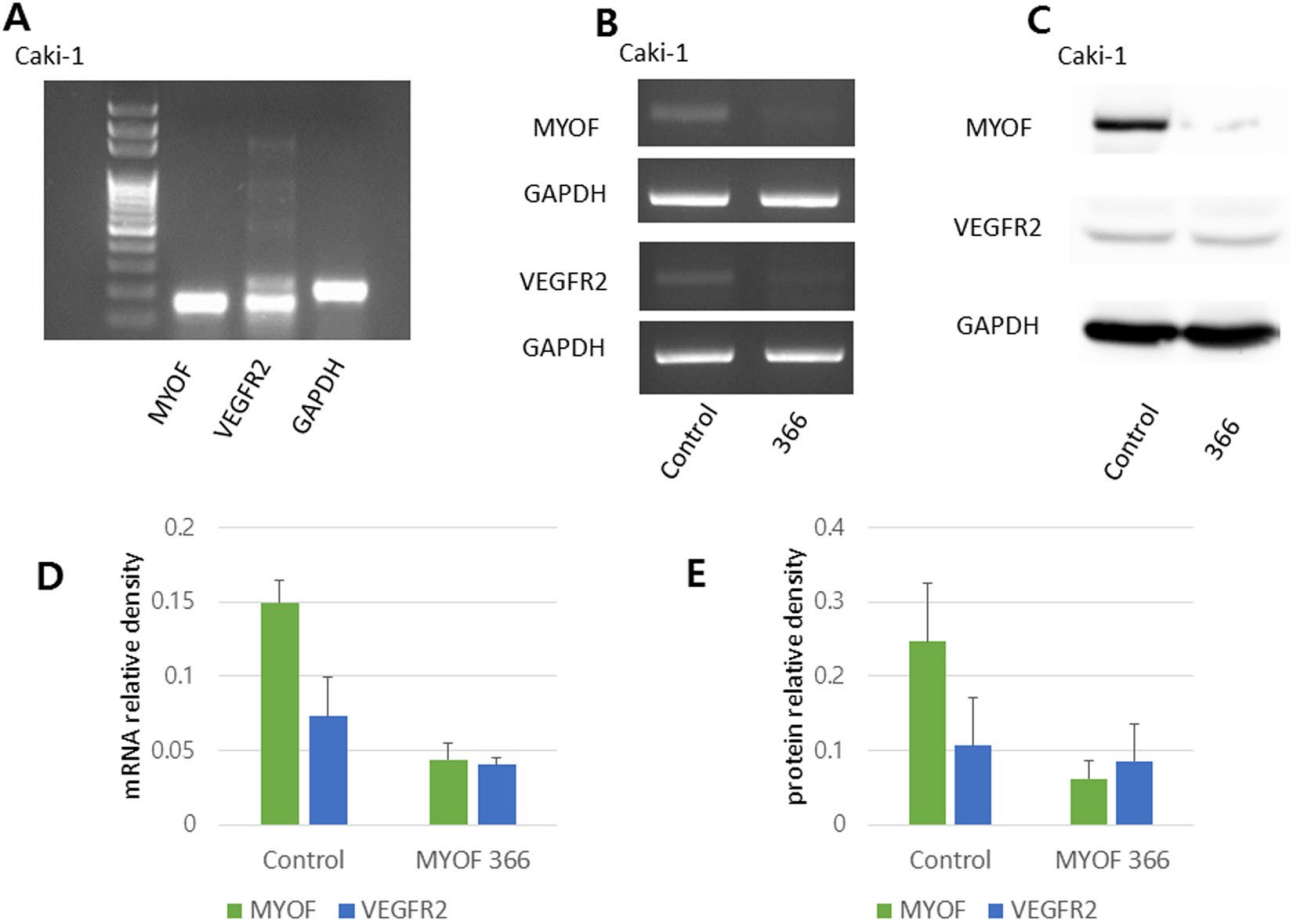
mRNA and protein expression of MYOF and VEGFR2 in Caki-1. **(A)** Total mRNA extracted from the human metastatic CCRCC cell line (Caki-1) was used to determine the mRNA levels of MYOF and VEGFR2 by quantitative PCR (qPCR). **(B)** Transfection of Caki-1 with MYO366 siRNAs revealed a decrease in mRNA and **(C)** protein levels of VEGFR2 compared with transfection with scrambled control siRNAs. Full-length gels and blots are presented in Supplementary Fig. S1. **(D)** The differences in relative VEGFR2 mRNA levels were larger than those in **(E)** VEGFR2 protein levels.

First, we evaluated the expression of MYOF and VEGFR2 mRNA in metastatic CCRCC. The mRNA levels of MYOF and VEGFR2 were estimated from total mRNA extracted from the human metastatic CCRCC cell line (Caki-1) using quantitative PCR (qPCR) (Fig. 2A). Western blot assays were used to determine the protein levels of MYOF and VEGFR2 in Caki-1 cells. Considering that Caki-1 is a metastatic CCRCC cell line, the identification of MYOF in Caki-1 was highly consistent with the result of our previous study on MYOF expression in CCRCC patients^3^.

### Loss of MYOF expression decreases both mRNA and protein levels of VEGFR2

Seventy-two hours of transfection of Caki-1 cells with siRNAs against bovine-human MYOF mRNA (MYO366) downregulated MYOF mRNA expression by up to 72% compared to the transfection of cells with scrambled control siRNAs. Transfection of Caki-1 with MYO366 siRNAs resulted in decreased mRNA (Fig. 2B) and protein levels (Fig. 2C) of VEGFR2 compared with transfection of cells with scrambled control siRNAs. In addition, differences in the relative mRNA levels of VEGFR2 (Fig. 2D) were larger than those in protein levels (Fig. 2E). This revealed that MYOF expression and VEGFR2 expression are associated not only at the transcriptional level but also at the translational level.

### MYOF silencing inhibits the VEGFR2-mediated downstream signaling pathway

**Figure 3:**
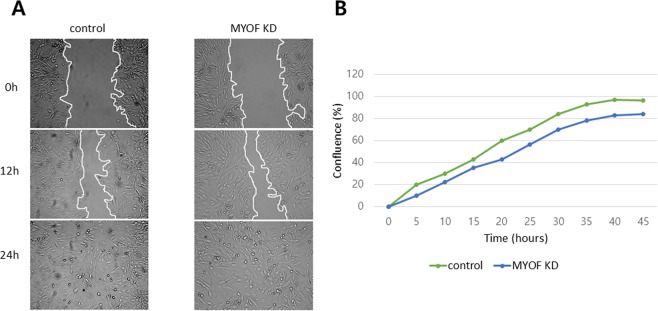
Wound healing assay. **(A)** Twenty-four hours of wound healing assay showed no significant difference between the MYOF silencing and the control group. **(B)** Cell confluence is different at 48 hours, with 83% in the MYOF-silenced group and 98% in the control group.

During the 12 hours of the wound healing assay, there were no significant differences between the MYOF-silenced group and the control group (Fig. 3A) as measured by ImageJ. This indicated that MYOF inhibition did not affect cell migration in metastatic CCRCC. However, cell confluence after 48 hours differed markedly and was 83% for the MYOF-silenced group and 98% for the control group (Fig. 3B), demonstrating that cell proliferation requires MYOF activity.

## Discussion

VEGFR2 is a receptor tyrosine kinase (RTK), and VEGFR2-mediated downstream signaling pathways are associated with cellular migration, proliferation and survival and endothelial cell permeability^7–9^. In endothelial cells, VEGFR2 forms complexes with MYOF and caveolin to avoid degradation^4^. Bernatchez *et al*. revealed that VEGFR2 forms a complex with dynamin-2 and MYOF in the plasma membrane of endothelial cells, which prevents VEGFR2 from degradation or ubiquitination^5^. Endosomes containing VEGFR or other RTKs continue to send signals or are rapidly degraded, depending on their internal components^10^. RTK internalization is a key factor that occurs during clathrin- or nonclathrin-mediated endocytosis (NEC) and determines the fate of RTKs^11^. Among the factors associated with NEC, caveolin is a specialized plasma membrane protein expressed in various types of mammalian cells and plays a major role in endocytosis, signal transduction, and oncogenesis^12,13^. Caveolin is found not only in endothelial cells but also in cancer cells; however, it plays contrasting roles in tumor progression and suppression^14,15^. Caveolin-2 (Cav-2) is known to be involved in CCRCC^14^. Cav-2, in association with miR-218, acts as a tumor promoter in renal cell carcinoma^14^. MYOF is a large protein containing a transmembrane domain and six C2 domains that contributes to plasma membrane repair, fusion, and endocytosis^5,16–19^. MYOF forms a complex with VEGFR2 to inhibit VEGFR2 degradation, as described above. In contrast, MYOF degrades and recycles EGFR by creating a complex with EGFR and caveolin^10^. Here, we assumed that complex formation by MYOF, Cav-2, and VEGFR2 may inhibit the degradation of VEGFR2 in metastatic CCRCC.

Wound healing assays have been widely used to measure cell migration activity^20^. Recently, a 384-well wound healing assay using various imaging techniques was used to detect underlying cellular processes apart from cell migration, including tissue reorganization and cell division^21^. In our study, there were almost no differences in cell migration between the control group and the MYOF knockdown group (Fig. 3A), as the slopes of the two graphs were similar. (Fig. 3B) Since different types of cancer cells show distinct patterns of metastatic progression driven by specific chemokine secretion and gene expression, MYOF silencing can result in diverse effects in various cells. As described above, Bernatchez PN *et al*. suggested that in endothelial cells, VEGFR2 forms complex with dynamin-2 or caveolin and MYOF to retain its angiogenetic roles. Loss of MYOF results in the lack of proliferation, migration, and nitric oxide release in endothelial cells and vascular tissues^5^. In breast cancer cell lines, MYOF forms a complex with EGFR to induce EGFR recycling. MYOF loss blocks EGF-induced cell migration and epithelial to mesenchymal transition^10^. Since Caki-1 is a metastatic CCRCC line, different cell surface receptors or tumor microenvironments may exist. Unlike that in endothelial cells or breast cancer cells, knockdown of MYOF in renal cell carcinoma cells may affect one of the VEGFR2-mediated downstream signaling pathways not related to tumor cell migration. Additionally, cell confluence was different after 48 hours (Fig. 3B). Analysis of cell confluence at 48 hours revealed lower confluence in the MYOF knockdown group than in the control group. In a previous study, VEGFR2 has been shown to form a complex with MYOF and activate the RAS oncogene, a signal-transducing protein, to induce signaling cascades, thereby maintaining the proliferation of cancer cells^6^. Similarly, in another previous *in vivo* study, VEGFR2 protein levels and VEGF-mediated vascular permeability were reduced in MYOF-deficient mice^4^. The RAS signaling cascade (RAS/RAF/MAP kinase) is involved in initiating events leading to cancer^22,23^. For example, BRAF, a member of the BAF family, was detected in up to 60% of tumor cells in malignant melanoma^23^. In our study, knockdown of MYOF in Caki-1 cells reduced proliferation without affecting migration. Among many intracellular signal transduction pathways, including p38 MAPK, NFAT, RACK1, SRC, PKB/AKT, and RAS, dysregulation of the RAS/RAF/MAP kinase cascade may alter nuclear gene transcription, thereby promoting CCRCC cell proliferation^7,24^.

By statistical analysis of CCRCC TMAs, we revealed that the positive intensity and high proportion of MYOF were significantly correlated with the negative intensity (p < 0.001) and low proportion (p < 0.001) of VEGFR2, respectively. In addition, Fuhrman’s nuclear grade ≥3 was significantly correlated with a high proportion of VEGFR2. Fuhrman’s nuclear grade was identified as an independent predictor of survival in CCRCC. Four pathological grades are considered within this approach. Bradley C *et al*. insisted that the most significant limitations in studying renal cell carcinoma are using the American Joint Committee on Cancer (AJCC) stage as a predictive factor and ignoring the histopathology of the disease^22^. More standardized nuclear and nucleolar criteria are therefore needed. Finally, the positive intensity of MYOF and the number of CD31-positive endothelial cells displayed a statistically significant correlation (p = 0.002). In this regard, evaluating MYOF and VEGFR2 expression might be an efficient alternative approach to predict CCRCC outcomes since both factors are associated with poor clinical outcomes in CCRCC based on multivariate analysis.

In our study, the difference in the relative levels of VEGFR2 mRNA (Fig. 2D) was greater than that in the levels of VEGFR2 protein (Fig. 2E). Hypothetically, in Caki-1 cells transfected with siRNAs (MYOF366), the mRNA and protein levels of MYOF in the cytoplasm are consistently reduced. Without MYOF, VEGFR2 protein starts to be degraded. To compensate for this loss, to rebalance VEGFR2 protein levels and to maintain cellular metastatic potential, the rate of VEGFR2 translation is increased, resulting in decreased VEGFR2 mRNA levels. Although the decrease in MYOF protein levels leads to VEGFR2 protein degradation, compensatory VEGFR2 translation might reduce the difference in the relative protein levels compared with the difference in mRNA levels between the MYOF-deficient and control groups. Previously, using immunoprecipitation, Bernatchez *et al*. proved that myoferlin siRNA (hMyof1-2) downregulated VEGFR2 expression in HUVECs^4^. However, they did not reveal an association between MYOF and VEGFR2 at the transcriptional level. To the best of our knowledge, this is the first study demonstrating the relevance of MYOF and VEGFR2 expression in metastatic CCRCC at both mRNA and protein levels.

One limitation of our study is that we excluded factors other than MYOF that may contribute to VEGFR2 degradation. We could not confirm the existence of caveolin-2 in the Caki-1 cell line since we adopted the data from previous studies^12,14,15^. Until now, there are only a few approaches for treating patients with metastatic CCRCC. Anti-VEGF therapy or immunotherapies are widely used^25–27^. Not until recently, a small molecule, WJ460, which targets and pharmacologically inhibits MYOF has been found to reduce breast cancer extravasation into the lung in an animal model^28^. Based on our study, therapies targeting MYOF combined with ramucirumab, a new anti-VEGFR therapy, might provide an efficient alternative approach for metastatic CCRCC to increase overall and disease-specific survival in patients. In conclusion, MYOF influences cellular proliferation in metastatic CCRCC by regulating VEGFR2 degradation.

## Materials and Methods

### Case selection

Specimens from 152 patients who underwent nephrectomy for CCRCC between January 2000 and December 2009 at the Gyeongsang National University Hospital, Jinju, Korea, were selected. Hematoxylin and eosin-stained sections on glass slides were reviewed by two pathologists. Electronic medical records were reviewed, and clinicopathological data, including sex, age, T stage, Fuhrman’s nuclear grade, recurrence, and follow-up period, were obtained. Cancer stages were determined according to the seventh edition of the American Joint Committee on Cancer (AJCC). The histological type and Fuhrman’s nuclear grade of the tumors were determined as per the fourth edition of the World Health Organization (WHO) classification. This study was approved by the institutional review board of Gyeongsang National University Hospital with a waiver of informed consent (GNUH-2015-12-001).

### Tissue microarray & immunohistochemistry

Representative hematoxylin and eosin-stained sections on glass slides containing prominent intratumoral regions from the 152 CCRCC specimens were chosen. Two cores were obtained from each representative paraffin block and transferred to recipient TMA blocks. Immunohistochemical staining was performed on the TMA blocks. Anti-MYOF monoclonal antibody (1:100 dilution, #ab76746, Abcam, UK), anti-VEGFR2 polyclonal antibody (1:50 dilution, #RB-1526-P1, Thermo Fisher Scientific, USA), and anti-CD31 monoclonal antibody (1:100 dilution, JC70, Cell Marque, USA) were used as the primary antibodies. The absolute number of positive CD31 endothelial cells was automatically counted using the Genie analysis tool (Leica Biosystems, Wetzlar, Germany).

### Cell culture & knockdown of MYOF

The CCRCC cell line Caki-1 (a metastatic cell line) was purchased from the Korean Cell Line Bank (Seoul, South Korea). Caki-1 cells were cultured in Dulbecco’s modified Eagle’s medium (DMEM, Gibco, #11995-065) supplemented with 10% fetal bovine serum (FBS, Gibco, #26140-079) and 1% penicillin-streptomycin (Corning, #30-002-CI) and incubated at 37 °C in an atmosphere containing 5% CO_2_. Caki-1 cells were cultured to 70–80% confluence in 60-mm dishes. The cells were transfected using Lipofectamine 3000 (Invitrogen, #L3000015) with human MYOF siRNAs (siMYOF, Bioneer, #1052366) or negative control scrambled siRNAs (Bioneer, #SN-1002) at a final concentration of 50 nM. After 24 hours of incubation, the cells were retransfected using the same protocol as described above. The cells were incubated for 72 hours before harvesting.

### Semiquantitative PCR

Total RNA was extracted using TRIzol reagent (Qiagen). Total RNA was quantified using a NanoDrop 2000 (Thermo Fisher Scientific), and 1 µg of total RNA was reverse transcribed to cDNA using a Maxime RT PreMix Kit (iNtRON, #25081). Equal amounts of synthesized cDNA were used for semiquantitative PCR using the Maxime PCR PreMix kit (iNtRON, #25025). Primers specific for MYOF (Bioneer, #P179687) and VEGFR2 (Bioneer, #P308687) were used. The primer sequences used for GAPDH are as follows: forward, 5′-GTC CAC CAC CCT GTT GCT GTA G-3′ and reverse, 5′-CAA GGT CAT CCA TGA CAA CTT TG-3′.

### Western blot analysis

Proteins were extracted using RIPA lysis buffer (Thermo Fisher Scientific, #89900) containing protease inhibitor cocktail (Thermo Fisher Scientific, #78430). The total protein concentration of each cell lysate was measured by the Bradford method using bovine serum albumin as a standard. Equal amounts of protein lysates (45 µg) were loaded on a denaturing polyacrylamide gel and then transferred to a nitrocellulose membrane. The primary antibodies used for immunoblotting were anti-MYOF (Abcam, #ab76746, UK), anti-VEGFR2 (Thermo Fisher Scientific, #RB-1526-P1, USA), and anti-GAPDH (Abcam, #ab8245, UK). Subsequently, the membranes were incubated with horseradish peroxidase-conjugated secondary antibodies and developed by enhanced chemiluminescence reaction (Thermo Fisher Scientific, #32109). Digital chemiluminescence images were captured by Fusion solo (Vilber).

### Wound healing and proliferation assays

Caki-1 cells were transfected with siMYOF as described above. Once the cells reached 100% confluence, a linear scratch wound was created using a 200 µl pipette tip. The cells were washed twice with PBS to remove detached cells. Then, the cells were incubated at 37 °C in an atmosphere containing 5% CO_2_, and the wounded area was monitored using JuLI Br (NanoEntek) and measured using ImageJ (NIH, USA). Caki-1 cells pretreated with siMYOF were seeded in 24-well plates at 10% confluence. The cell confluence was monitored using JuLI Br (NanoEntek).

## Supplementary information

supplemetal dataset1, dataset2

## References

[1] Zhang W, Zhou P, Meng A, Zhang R, Zhou Y (2018). Down‐regulating Myoferlin inhibits the vasculogenic mimicry of melanoma via decreasing MMP‐2 and inducing mesenchymal‐to‐epithelial transition. J Cell Mol Med.

[2] Barnhouse VR (2018). Myoferlin regulates epithelial cancer cell plasticity and migration through autocrine TGF-β1 signaling. Oncotarget.

[3] Rademaker, Costanza, Anania, Agirman, Maloujahmoum, Di Valentin, Goval, Bellahcène, Castronovo, Peulen (2019). Myoferlin Contributes to the Metastatic Phenotype of Pancreatic Cancer Cells by Enhancing Their Migratory Capacity through the Control of Oxidative Phosphorylation. Cancers.

[4] Bernatchez PN (2007). Myoferlin regulates vascular endothelial growth factor receptor-2 stability and function. J. Biol. Chem..

[5] Bernatchez PN, Sharma A, Kodaman P, Sessa WC (2009). Myoferlin is critical for endocytosis in endothelial cells. American Journal of Physiology-Cell Physiology.

[6] Song DH (2017). Prognostic role of myoferlin expression in patients with clear cell renal cell carcinoma. Oncotarget.

[7] Abhinand CS (2016). VEGF-A/VEGFR2 signaling network in endothelial cells relevant to angiogenesis. Journal of cell communication and signaling.

[8] Koch S, Claesson-Welsh L (2012). Signal transduction by vascular endothelial growth factor receptors. Cold Spring Harb Perspect..

[9] Chekhonin VP (2013). VEGF in tumor progression and targeted therapy. Current cancer drug targets.

[10] Turtoi A (2013). Myoferlin is a key regulator of EGFR activity in breast cancer. Cancer Res..

[11] Grant BD, Donaldson JG (2009). Pathways and mechanisms of endocytic recycling. Nature reviews Molecular cell biology.

[12] Yamasaki T (2013). MicroRNA-218 inhibits cell migration and invasion in renal cell carcinoma through targeting caveolin-2 involved in focal adhesion pathway. J. Urol..

[13] Ariotti N, Parton RG (2013). SnapShot: caveolae, caveolins, and cavins. Cell.

[14] Gupta R, Toufaily C, Annabi B (2014). Caveolin and cavin family members: dual roles in cancer. Biochimie.

[15] Campbell L, Gumbleton M, Griffiths DFR (2003). Caveolin-1 overexpression predicts poor disease-free survival of patients with clinically confined renal cell carcinoma. Br. J. Cancer.

[16] Lek A (2012). Ferlins: regulators of vesicle fusion for auditory neurotransmission, receptor trafficking and membrane repair. Traffic.

[17] Cipta S, Patel HH (2009). Molecular bandages: inside-out, outside-in repair of cellular membranes.Focus on “Myoferlin is critical for endocytosis in endothelial cells” Am J Physiol. Cell Physiol..

[18] Doherty KR (2008). The endocytic recycling protein EHD2 interacts with myoferlin to regulate myoblast fusion. J. Biol. Chem..

[19] Ellis JA (2003). Cell biology: Patches for wounded muscle. Nature.

[20] Lampugnani, M. G. Cell migration into a wounded area *in vitro*. In: Adhesion Protein Protocols. 177–182 (Springer, 1999). 10.1385/1-59259-258-9:177 10098136

[21] Yarrow JC (2004). A high-throughput cell migration assay using scratch wound healing, a comparison of image-based readout methods. BMC biotechnology.

[22] Leibovich BC (2018). Predicting oncologic outcomes in renal cell carcinoma after surgery. Eur. Urol..

[23] Kumar, V. *et al*. Robbins and Cotran pathologic basis of disease, eighth edition. 280–283 (Elsevier health sciences, 2010).

[24] Koch S (2011). Signal transduction by vascular endothelial growth factor receptors. Biochem. J..

[25] Choueiri TK (2008). Efficacy of sunitinib and sorafenib in metastatic papillary and chromophobe renal cell carcinoma. J. Clin. Oncol..

[26] Shojaei F (2012). Anti-angiogenesis therapy in cancer: current challenges and future perspectives. Cancer Lett..

[27] Belldegrun AS (2013). ARISER: A randomized double blind phase III study to evaluate adjuvant cG250 treatment versus placebo in patients with high-risk ccRCC—Results and implications for adjuvant clinical trials. Journal of Clinical Oncology.

[28] Zhang T (2018). A small molecule targeting myoferlin exerts promising anti-tumor effects on breast cancer. Nat Commun.

