# Supplementary material for: Myoferlin silencing inhibits VEGFR2-mediated proliferation of metastatic clear cell renal cell carcinoma
Source: Sci Rep. 2019 Sep 2;9:12656. doi: 10.1038/s41598-019-48968-7 (PMC6718427; doi:10.1038/s41598-019-48968-7)
Supplement: Supplementary file 1 — supplemetal dataset1, dataset2 [file 41598_2019_48968_MOESM1_ESM.pdf]

SUPPLEMENTAL INFORMATION

MANUSCRIPT TITLE: Myoferlin silencing inhibits VEGFR2-mediated proliferation of metastatic clear cell renal cell carcinoma.

Hyo Jung An[1], Dae Hyun Song[1, 2, 3] ,Hyun Min Koh[1], Yu-Min Kim[3] ,Gyung Hyuck Ko [2, 3, 4], Jeong-Hee Lee [2, 3,4], Jong Sil Lee [2, 3,4], Jung Wook Yang[4], Min Hye Kim[4], Deok Ha Seo[5], Se Min Jang[6], and Dong Chul Kim[2, 3, 4]

[1] Department of pathology, Gyeongsang National University Changwon Hospital;[2] Gyeongsang National University School of Medicine; [3]Gyeongsang Institute of Health Science; [4]Department of pathology, Gyeongsang National University Hospital, Jinju, Korea; [5] Department of urology, Gyeongsang National University Changwon Hospital; [6]Department of pathology, Konyang University Hospital

SUPPLEMENTAL DATASET 1

MYOF

VEGFR2

GAPDH

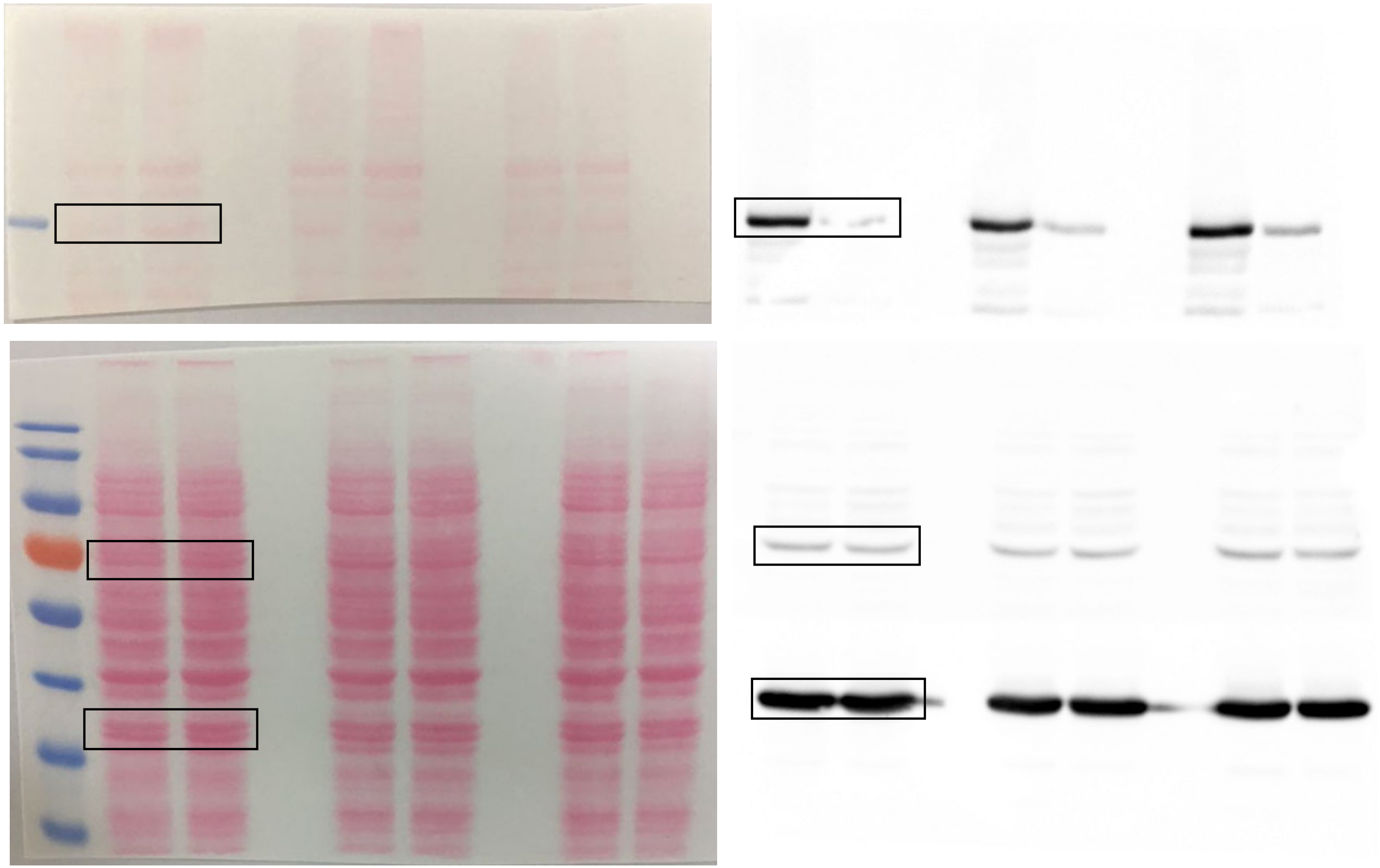

Figure S1.Original immunoblots used to crop the gel bands for Figure 2C.

SUPPLEMENTAL DATASET 2

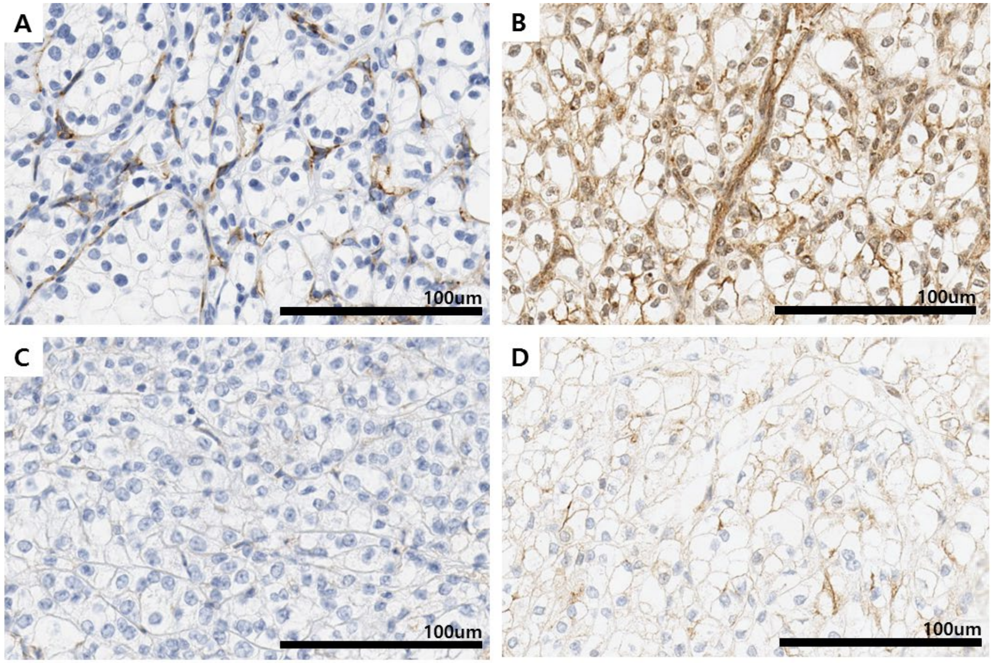

Figure S2. Representative images of cores with CD31-positive endothelial cells and corresponding MYOF expression (A) A core with more than 5000 CD31-positive endothelial cells. (B) The corresponding core with positive MYOF intensity. (C) A core with Less than 5000 CD31-positive endothelial cells. (D) The corresponding core with negative MYOF intensity.
